# Correlation between Serum IL-35 Levels and Bone Loss in Postmenopausal Women with Rheumatoid Arthritis

**DOI:** 10.1155/2019/9139145

**Published:** 2019-08-26

**Authors:** Yuxuan Li, Lutian Yao, Siyan Liu, JishengWu, Liping Xia, Hui Shen, Jing Lu

**Affiliations:** ^1^Department of Rheumatology and Immunology, The First Affiliated Hospital of China Medical University, 155 Nanjing North Street, Heping District, Shenyang 110001, China; ^2^Department of Sports Medicine and Joint Surgery, The First Affiliated Hospital of China Medical University, 155 Nanjing North Street, Heping District, Shenyang 110001, China

## Abstract

**Objective:**

IL-35 was reported as a crucial anti-inflammatory cytokine and could efficiently regulate bone metabolism in murine collagen-induced arthritis model. However, the relationship between IL-35 and bone health in human rheumatoid arthritis (RA) has not been clarified. In this study, the aim was to explore the correlations between IL-35 and bone loss in postmenopausal women with RA.

**Methods:**

The study included 76 postmenopausal women with RA and 53 healthy postmenopausal women as healthy controls (HCs). Serum IL-35 levels were detected by enzyme-linked immunosorbent assay. Bone mineral density (BMD) at lumbar spine 1-4 and at total hip was measured using dual-energy X-ray absorptiometry. Alkaline phosphatase (ALP), *β*-isomerised carboxy-terminal cross-linking telopeptide of type I collagen (*β*-CTX), and 25-(OH) VitD_3_ were measured by turbidimetric inhibition immunoassay.

**Results:**

Serum IL-35 levels were increased compared with HCs, and it positively correlated with BMD and 25-(OH) VitD_3_ and negatively correlated with *β*-CTX in postmenopausal women with RA. Furthermore, serum IL-35 levels in the increased ALP group were higher than those in the normal ALP group.

**Conclusions:**

IL-35, an important anti-inflammatory cytokine, may participate in the pathogenesis of bone loss in postmenopausal women with RA.

## 1. Introduction

Bone loss is a severe complication in RA patients which exhibits increased fracture risk, morbidity, and mortality. Dual-energy X-ray absorptiometry (DXA) assessments have shown a rather high prevalence of bone loss in RA patients with merely a few weeks of symptom duration and no exposure to glucocorticoids [1, 2]. Up to 25% of recently diagnosed patients with RA had bone loss before treatment and 11% had developed to osteoporosis [3]. It has been well demonstrated that bone loss links to the presence of cytokines, which regulates reciprocal interactions between osteoclasts, osteoblasts, and immune system cells, which finally significantly influences bone homeostasis.

Interleukin- (IL-) 35 is a new member of the IL-12 family, consisting of Epstein-Barr virus-inducible gene 3 (Ebi3) subunit and p35 (IL-12*α*) subunit [4]. In 2007, Collison et al. discovered that IL-35 is secreted by regular T cells (Tregs) and inhibits the function of T effector cells [5]. Studies have confirmed the efficacy of IL-35 in autoimmune diseases such as rheumatoid arthritis, systemic lupus erythematosus, and inflammatory bowel disease [6–8]. Our group also demonstrated that the treatment of IL-35 could upregulate the ratio of osteoprotegerin and receptor activator of nuclear factor *κ*B ligand in fibroblast-like synoviocytes from collagen-induced arthritis mice, indicating its potential role in RA, especially with bone loss [9].

However, information on IL-35 from postmenopausal women with RA is scant. Therefore, this study was to measure the serum levels of IL-35 and its association with bone loss-related parameters including bone mineral density (BMD), bone turnover markers (BTMs), and 25(OH) VitD_3_ in postmenopausal women with RA.

## 2. Materials and Methods

### 2.1. Patients and Healthy Controls (HCs)

A total number of 76 patients who satisfied the 2010 American College of Rheumatology RA criteria were recruited in this study [10]. All postmenopausal women in the case groups and HCs' age were over 45 years, and postmenopausal status was defined by the absence of menstrual periods for at least 12 months. Patients were carefully excluded with definitive diagnoses other than RA. Patients with previous history of other secondary bone loss (e.g., diabetes and hyperparathyroidism) and history of use of agents which interfere with BMD (calcium or vitamin D treatment, glucocorticoids, sex hormones, or other bone active drugs) were excluded. Clinical parameters regarding RA including erythrocyte sedimentation rate (ESR), C-reactive protein (CRP), rheumatoid factor (RF), anticyclic citrullinated peptide antibodies (ACPA), swollen joint count (SJC), tender joint count (TJC), and medication types were recorded. Clinical parameters and DXA values were also obtained from 53 age-matched healthy postmenopausal women as HCs. Written informed consent was provided by all subjects, and the study was approved by the ethics committee of the First Affiliated Hospital of China Medical University and was conducted according to the Declaration of Helsinki.

### 2.2. BMD Assessments

BMD assessment at the lumbar spine 1-4 (L1-L4) and total hip was performed using the same DXA equipment. According to the World Health Organization guidelines, osteoporosis was defined as having a T score of −2.5 or less at any site. Osteopenia was defined as having a T score between −1 and −2.5 at any site [11].

### 2.3. BTMs and Vitamin D Assessments

BTMs including *β*-isomerised carboxy-terminal cross-linking telopeptide of type I collagen (*β*-CTX) and alkaline phosphatase (ALP) were chosen to evaluate the bone remodeling process. *β*-CTX was used as the bone resorption marker. ALP was used as the bone formation marker. 25-(OH) VitD_3_ was choose to present vitamin D statue. BTMs and 25-(OH) VitD_3_ were measured by turbidimetric inhibition immunoassay.

### 2.4. Serum IL-35 Levels Measurement

Serum IL-35 levels were measured using enzyme-linked immunosorbent assay (ELISA) kits (eBioscience, San Diego, CA, USA) according to the manufacturer's protocol. The optical density was measured at 450 nm using an automatic ELISA reader.

### 2.5. Statistical Analysis

Data were presented as mean ± standard error (SE) or median (interquartile range (IQR)) if continuous and as counts and percent if categorical. Two variables from the study were analyzed by the Student's *t*-test with a parametric distribution or the Mann–Whitney *U* test with a nonparametric distribution. Pearson's or Spearman's correlation coefficient was used to test the correlations between two variables. All analyses were performed by using SPSS 17.0 (SPSS Inc., Chicago, IL) and GraphPad prism 6 software. Differences of *p* < 0.05 were considered significant.

## 3. Results

### 3.1. Characteristics of RA and HCs

The postmenopausal women with RA had a mean ± SE age of 52.5 ± 2.4 years with a mean disease duration of 4.8 months. No one was treated with glucocorticoid and biological therapy. Osteoporosis was observed in 19.7% of patients. In contrast, osteopenia was much more common, observed in 57.9% of patients. Nine (11.8%), 13 (17.1%), and 54 (71.1%) patients had remission (DAS28 < 3.2), moderate (3.2 ≤ DAS28 ≤ 5.1), and high (DAS28 > 5.1) disease activity as assessed using DAS28 score based on ESR, respectively (Table 1).

### 3.2. Serum Levels of IL-35 in relation to Bone Loss in Patients with RA

**Figure 1:**
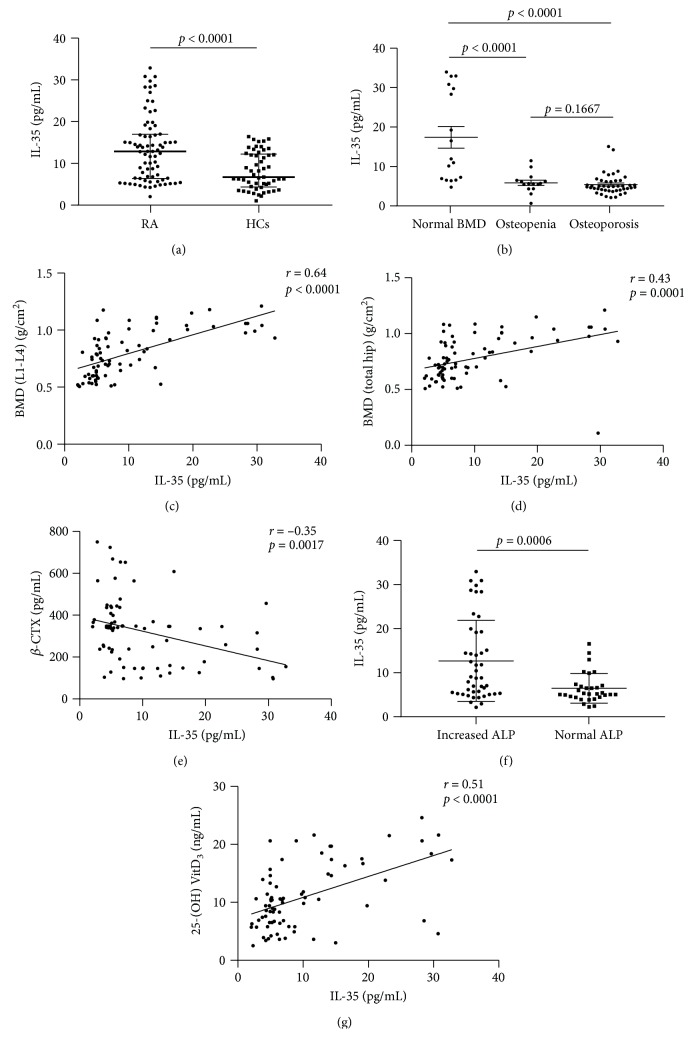
(a) Serum IL-35 levels in patients with RA and HCs. (b) Serum IL-35 levels in RA patients with normal BMD, osteopenia, and osteoporosis. (c–g) Correlation between serum IL-35 levels and BMD at L1-L4, BMD at total hip, *β*-CTX, ALP, and 25-(OH) VitD_3_. Abbreviations: IL: interleukin; RA: rheumatoid arthritis; HCs: healthy controls; BMD at L1-L4: bone mineral density at lumbar spine 1-4; *β*-CTX: *β*-isomerised carboxy-terminal cross-linking telopeptide of type I collagen; ALP: alkaline phosphatase.

Serum IL-35 levels in RA patients (12.9 (6.4-16.9) pg/mL) were higher than those in HCs (6.7 (4.3-12.2) pg/mL) (*p* < 0.0001) (Figure 1(a)). The serum levels of IL-35 in patients with normal bone mass was significantly higher compared to osteopenia and osteoporosis patients (*p* < 0.0001, *p* < 0.0001, respectively) (Figure 1(b)).

Serum levels of IL-35 had a positive correlation with BMD at L1-L4 (*r* = 0.64, *p* < 0.0001) and BMD at total hip (*p* = 0.43, *p* = 0.0001) (Figures 1(c) and 1(d)).

Serum levels of IL-35 had a negative correlation with *β*-CTX (*r* = −0.35, *p* = 0.0017) (Figure 1(e)).

Serum levels of IL-35 did not correlate with ALP (*r* = 0.2, *p* = 0.077). However, serum IL-35 levels in increased ALP group were higher than normal ALP group (*p* = 0.0006) (Figure 1(f)).

Serum levels of IL-35 had a positive correlation with 25-(OH) VitD_3_ (*r* = 0.51, *p* < 0.0001) (Figure 1(g)).

### 3.3. Serum Levels of IL-35 in relation to BMD: Multivariate Linear Regression Analysis

Considering that the *T* score and *Z* score did not statistically differ between patients with RA and HCs, we established a multivariate model to explore the covariates independently associated with BMD. Main covariates considered for entry were disease duration, ESR, CRP, DAS28-ESR, RF, ACPA, *β*-CTX, ALP, 25-(OH) VitD_3_, and serum IL-35 levels. IL-35 maintained significant in BMD at L1-L4 and total hip, indicating that serum IL-35 levels are significantly associated with BMD (Table 2).

## 4. Discussion

In our study, information on bone loss in RA was from postmenopausal women. In addition, since glucocorticoid utility has conventionally been correlated with adverse impact on bone health, the patients enrolled in our study were all not exposed with glucocorticoid. The results showed that serum IL-35 levels were significantly increased in postmenopausal women with RA as compared to HCs, suggesting an immunoregulatory role of IL-35 in RA, especially in postmenopausal women. Considering the anti-inflammatory properties of IL-35, it could be highly hypothesized that upregulation of serum IL-35 levels is due to the immune negative feedback response to excessive inflammatory factors such as tumor necrosis factor-*α* and IL-6.

BMD can reflect bone strength, and it is considered to be the gold standard for the diagnosis of bone loss. In our study, serum IL-35 levels were positively correlated with BMD at L1-L4 and total hip. Furthermore, the multiple linear regression analysis suggested that the relationships between serum IL-35 levels and BMD were not changed. This association remained significant after adjustment suggesting a significant effect of IL-35 on BMD in RA patients, suggesting that serum IL-35 levels might be a viable option for monitoring the extent of bone mass in postmenopausal women with RA.

The information BMD provided is nondynamic and not sensitive enough to detect early bone loss. BTMs can reflect the structured status of trabecular bone and provide helpful information regarding the bone remodeling process. Furthermore, BTMs are also useful for selecting patients who would respond well to antiosteoporotic treatment. Under estrogen deficiency, serum IL-35 levels are negatively correlated with *β*-CTX. We did not find a correlation between serum IL-35 levels and ALP levels. However, serum IL-35 levels in the increased ALP group were higher than those in the normal ALP group. This may explain that total ALP lacks specificity. Serum bone-specific alkaline phosphatase (BALP), which is expressed on the surface of osteoblasts, should be measured for the improvement of the study. Previous study showed that BALP synthesis positively correlated with bone formation [12]. It is well demonstrated that bone resorption and bone formation are both increased in postmenopausal bone loss. However, the extent of augmented bone resorption exceeds that of increased bone formation, which results in an imbalance between bone formation and bone resorption in favor of bone resorption [13, 14]. The main limitation in our study is that there is no data on articular bone erosion which represents localized bone loss. Measurement of joint damage with special attention given to juxta-articular bone erosions such as Sharp's score using imaging technologies will be needed to explore the correlation between IL-35 and localized bone loss in RA patients.

Vitamin D is widely used to treat patients with osteoporosis. It is well acknowledged not only for its classical role in bone metabolism but also for modulation of innate and adaptive immune system. In our study, blood samples of RA and HCs were collected at the same time. None of the subjects had ever used vitamin D supplement. Meanwhile, in the current study, we also found that RA disease activity was negatively correlated with vitamin D levels (data not shown), which coincides with the previous study [15]. However, we did not include data on physical activity levels in the analysis that influence vitamin D levels. In our study, increased serum IL-35 levels were associated with increased 25(OH) VitD_3_, indicating IL-35 may play an important role in bone health and immune system.

## 5. Conclusion

This study demonstrates for the first time that serum IL-35 levels are associated with bone loss and highlights that low BMD, high bone metabolism, and low vitamin D statue are still significant problems in postmenopausal women with RA, suggesting that IL-35 may represent a novel therapeutic target for RA, especially for RA with bone loss.

## Figures and Tables

**Table 1:** Clinical parameters of RA and HCs.

Characteristics	RA (*n* = 76)	HCs (*n* = 53)	*p* value
Demographics			
Age, years, mean ± SE	52.5 (2.4)	53.3 (2.8)	0.56
Menopause, years, mean ± SE	48.5 (2.1)	49.0 (2.0)	0.39
Duration of postmenopausal period, years, mean ± SE	8.9 (2.4)	8.2 (2.2)	0.41
Disease duration, months, median (IQR)	4.8 (0.7-7.0)	—	
Disease characteristics			
ESR, mm/h, mean ± SE	68.4 (2.7)	—	
CRP, mg/L, mean ± SE	44.0 (3.9)	—	
TJC, mean ± SE	9 (1.2)	—	
SJC, mean ± SE	6 (1.1)	—	
RF (+), *n* (%)	42 (55.3)	—	
ACPA (+), *n* (%)	53 (69.7)	—	
DAS28-ESR	6.6 (1.2)	—	
Medications			
HCQ, *n* (%)	36 (47.4)	—	
MTX, *n* (%)	18 (23.7)	—	
LEF, *n* (%)	16 (21.1)	—	
TG, *n* (%)	16 (21.1)	—	
DXA			
Normal, *n* (%)	17 (22.4)	17 (32.1)	<0.01
Osteopenia, *n* (%)	44 (57.9)	27 (50.9)	0.04
Osteoporosis, *n* (%)	15 (19.7)	9 (17.0)	0.04
Lumbar spine (L1-L4)			
BMD, g/cm^2^, mean ± SE	0.8 (0.3)	1.0 (0.3)	0.03
T score, mean ± SE	-2.2 (0.4)	-0.9 (0.4)	0.07
Z score, mean ± SE	-1.1 (0.4)	-0.5 (0.4)	0.06
Total hip			
BMD, g/cm^2^, mean ± SE	0.8 (0.3)	1.0 (0.4)	0.04
*T* score, mean ± SE	-1.3 (0.4)	-0.9 (0.4)	0.07
*Z* score, mean ± SE	-1.2 (0.3)	-0.6 (0.3)	0.06
BTMs			
Serum *β*-CTX, pg/mL, median (IQR)	340.3 (181.1-394.1)	123.6 (111.2-218.2)	<0.0001
Serum ALP, U/L, median (IQR)	78.0 (66.2-96.5)	60.1 (42.0-81.5)	0.1
Serum 25-(OH) VitD_3_, ng/mL median (IQR)	10.1 (6.4-14.8)	41.8 (8.1-13.3)	<0.0001

Abbreviations: ESR: erythrocyte sedimentation rate; CRP: C-reaction protein; TJC: tender joint count; SJC: swollen joint count; DAS28-ESR: disease activity score in 28 joints with sedimentation; RF: rheumatoid factor; ACPA: anticyclic citrullinated peptide antibodies; *β*-CTX: C-terminal cross-linked telopeptides of type I collagen; BMD: bone mineral density; HCQ: hydroxychloroquine; MTX: methotrexate; LEF: leflunomide; TG: tripterygium glycosides.

**Table 2:** Multivariate linear regression analysis of BMD.

Covariates	BMD					
	Lumbar spine (L1-L4)			Total hip		
	OR	95% CI	*p* value	OR	95% CI	*p* value
Disease characteristics						
Disease duration	0.010	-0.036-0.038	0.096	0.015	-0.033-0.035	0.095
ESR	-0.037	-0.002-0.002	0.075	-0.063	-0.001-0.002	0.063
CRP	-0.113	-0.002-0.001	0.315	-0.114	-0.002-0.001	0.339
DAS28-ESR	-0.258	-0.070-0.012	0.086	-0.301	-0.067-0.008	0.122
RF	-0.162	-0.007-0.004	0.065	-0.037	-0.005-0.0005	0.092
ACPA	-0.250	0.000-0.000	0.089	-0.300	0.000-0.000	0.097
BTMs						
*β*-CTX	-0.165	0.000-0.000	0.021	-0.075	0.000-0.000	0.050
ALP	0.063	-0.003-0.002	0.059	0.032	-0.002-0.002	0.079
25-(OH) VitD_3_	0.134	-0.003-0.013	0.082	0.032	-0.003-0.011	0.093
IL-35	0.463	-0.009-0.033	0.026	0.254	-0.014-0.025	0.025
Medications						
HCQ	0.156	-0.157-0.030	0.181	0.065	-0.109-0.063	0.596
MTX	0.027	-0.101-0.126	0.825	0.026	-0.093-0.115	0.836
LEF	0.075	-0.090-0.164	0.563	0.126	-0.062-0.171	0.356
TG	0.074	-0.081-0.154	0.534	0.213	-0.016-0.199	0.093

BMD adjusted for disease duration, ESR, CRP, DAS28-ESR, RF, ACPA, *β*-CTX, ALP, IL-35, 25-(OH) VitD_3_, and medications. Abbreviations: BMD: bone mineral density; ESR: erythrocyte sedimentation rate; CRP: C-reaction protein; DAS28-ESR: disease activity score in 28 joints based on erythrocyte sedimentation rate; RF: rheumatoid factor; ACPA: anticyclic citrullinated peptide antibodies; BTMs: bone turnover markers; *β*-CTX: *β*-isomerised carboxy-terminal cross-linking telopeptide of type I collagen; ALP: alkaline phosphatase; IL: interleukin; HCQ: hydroxychloroquine; MTX: methotrexate; LEF: leflunomide; TG: tripterygium glycosides; OR: odds ratio; CI: confidence interval.

## Data Availability

The data used to support the findings of this study are available from the corresponding author upon request.
